# LONG-TERM EFFECTS OF AN INTENSIVE LIFESTYLE INTERVENTION ON NUTRITIONAL RISK IN OLDER ADULTS WITH DIABETES

**DOI:** 10.1093/geroni/igae098.0388

**Published:** 2024-12-31

**Authors:** Denise Houston, Andrea Anderson, Charles Semelka, Kathleen Hayden, Peter Huckfeldt, Haiying Chen, Lynne Wagenknecht, Mark Espeland

**Affiliations:** Wake Forest University School of Medicine, Winston Salem, North Carolina, United States; Wake Forest University School of Medicine, Winston-Salem, North Carolina, United States; Wake Forest University School of Medicine, Winston-Salem, North Carolina, United States; Wake Forest University School of Medicine, Winston-Salem, North Carolina, United States; University of Minnesota School of Public Health, Minneapolis, Minnesota, United States; Wake Forest University School of Medicine, Winston-Salem, North Carolina, United States; Wake Forest University School of Medicine, Winston-Salem, North Carolina, United States; Wake Forest University School of Medicine, Winston-Salem, North Carolina, United States

## Abstract

Malnutrition is common in older adults and is associated with morbidity, mortality and decreased quality of life. We examined the post-intervention impact of the Look AHEAD intensive lifestyle intervention (ILI) on nutritional risk – the presence of risk factors such as food insecurity, poor appetite, and low food intake which can lead to malnutrition – in older adults with type 2 diabetes and overweight/obesity in the Look AHEAD Aging study (~9 years after the intervention was terminated). Nutritional risk scores (range 0-21) were calculated from the DETERMINE checklist, a 10-item questionnaire of factors linked to poor nutrition in older age, and categorized as low (0-2), moderate (3-5) and high (6+). Multinomial logistic regression was used to examine the association between intervention assignment, post-intervention weight change, unintentional weight loss, and current BMI with nutritional risk adjusted for current age, sex, race/ethnicity, and education in 1032 participants. Forty percent of participants were at high nutritional risk and 48% at moderate nutritional risk. There was no association between prior assignment to ILI (p=0.65) or post-intervention weight loss or gain of ≥5% (p=0.09) and nutritional risk. However, unintentional weight loss of ≥10 lbs over the past year was associated with high nutritional risk (OR [95% CI]: 2.18 [1.16-4.09]) and current BMI of ≥30 kg/m2 was similarly associated with both moderate (1.62 [1.01-2.60]) and high (1.68 [1.03-2.74]) nutritional risk. In conclusion, current BMI and recent unintentional weight loss, but not past assignment to ILI or post-intervention weight loss/gain, were associated with nutritional risk.

